# Determinants of personal protective measures use to prevent the spread of respiratory infectious diseases in early 2024, Canada: a cross-sectional study

**DOI:** 10.3389/fpubh.2026.1943332

**Published:** 2026-10-27

**Authors:** Katie Graham, Khater A. Idriss, Ruth Davy, Brittany Blouin, Paniz Tavakoli, Toju Ogunremi

**Affiliations:** 1 Public Health Measures Guidance Division, Centre for Emerging Respiratory Infections and Pandemic Preparedness (CERIPP), Public Health Agency of Canada, Ottawa, ON, Canada; 2 Department of Epidemiology and Biostatistics, Schulich School of Medicine & Dentistry, Western University, London, ON, Canada; 3 Public Engagement, Research and Analysis Directorate, Communications and Public Affairs Branch, Health Canada, Ottawa, ON, Canada; 4 Behavioral Science Office, Science and Policy Integration Branch, Public Health Agency of Canada, Ottawa, ON, Canada; 5 Impact Canada, Privy Council Office, Ottawa, ON, Canada

**Keywords:** health communication, health promotion, non-pharmaceutical intervention, pandemic preparedness, perceived effectiveness, public health measure, risk perception, risk reduction behavior

## Abstract

This study explored how people in Canada make decisions regarding the use of personal protective measures (PPMs) to protect themselves and others from respiratory infectious diseases (RIDs). It examined patterns and perceptions of PPM use post-COVID-19 pandemic and potential determinants of ongoing PPM use. A cross-sectional survey with 6,611 respondents, selected from online panels to represent the Canadian population, was conducted in February 2024. Descriptive statistics and multivariate linear regression models were used to describe and understand the potential determinants of PPM use. While PPMs were generally perceived as effective, their adoption varied. Hand washing and covering coughs and sneezes were common hygiene habits post-pandemic. Cleaning high-touch areas and improving ventilation had more barriers to uptake. Staying home when sick, while perceived as effective by almost all respondents, was reported by only two thirds of those sick in the past 3 months. Mask-wearing in indoor public settings was viewed as the least effective and least adopted PPM. In each tested PPM’s multivariate linear regression model (adjusted *R*^2^ ranged from 0.216–0.407 across 6 models) several factors were associated with PPM use, with perceived effectiveness of PPMs (*p* < 0.001) and concern with getting sick (*p* < 0.001) consistently associated with most PPMs. Commonly cited reasons for not using PPMs included them not being mandatory and having barriers to their implementation. Results from this study can help inform supports and public health communication strategies for PPM use during current and future seasonal RID outbreaks and during pandemics.

## 1 Introduction

Non-pharmaceutical public health measures that can be used by individuals, otherwise known as personal protective measures (PPMs), are important public health interventions for reducing the transmission of respiratory infectious diseases (RIDs) such as COVID-19, influenza, and respiratory syncytial virus (RSV).

PPMs include staying home when sick, wearing a well-fitting mask, practicing hand hygiene, improving ventilation, and covering coughs and sneezes. There exists a considerable body of literature regarding the efficacy and/or effectiveness of these interventions in reducing RID transmission in community settings (1–6).

Despite the established effectiveness of many of these PPMs, their adoption has been found to vary widely across populations, influenced by sociodemographic factors, personal attitudes, perceived risks, and ease of adoption (7, 8). Previous research, primarily conducted during the acute phase of various epidemics, identified that individuals who were older, who identified as women, who had higher trust in governments or authorities, who perceived the illness as a serious threat, and who relied on traditional news media for information, were more likely to report adopting PPMs during an epidemic (7–10).

Some additional drivers of PPM adherence in the literature included access to information, previous knowledge about diseases, and trust in healthcare professionals and family or friends (11, 12). Mental health and emotional drivers of behavior have also been explored, with studies suggesting that anxiety and ability to cope have potential impacts on adherence (7, 12, 13).

While the determinants of PPM use during a pandemic or outbreak have been frequently studied, less is known about variations in PPM use and drivers in the period after the acute phase of an outbreak. Studies that have examined protective behaviors outside of an outbreak have been focused on knowledge and behaviors linked to outbreak specific RIDs, were conducted closer to resurgent waves, or were focused on localized public health objectives (14, 15). Although these studies provide important evidence, they do not look at drivers across the range of individual PPMs, rather they focus on broader adoption. Given the scale of the COVID-19 pandemic, this period may have shifted behavior patterns in ways that differ from previous epidemics. Understanding the drivers of PPM use is vital for developing public health interventions, supports, and communication strategies for seasonal RIDs and future outbreaks.

This research set out to describe PPM adoption used to reduce RID transmission among individuals in Canada in early 2024, after the acute phase of the COVID-19 pandemic and during cold and flu season. In May 2023, the World Health Organization determined that COVID-19 no longer constituted a Public Health Emergency of International Concern and recommended a transition toward the long-term management of SARS-CoV-2 as an ongoing health issue (16). At the time of the survey, Canadian national surveillance data indicated that SARS-CoV-2 test positivity had been declining since mid-December 2023 and case counts were lower than in the previous years of the pandemic (17). Within this context, the study explored motivators, drivers, and the barriers associated with reported PPM use. The objective of this study was to investigate patterns and key determinants of PPM use.

## 2 Methods

### 2.1 Study design and participants

The cross-sectional online survey, commissioned by the Public Health Agency of Canada, was conducted by market research firm Abacus Data between February 15 and 28, 2024. This study was reviewed by the Government of Canada Public Opinion Research Directorate (#085–23).

A total of 6,611 individuals aged 12 and older were recruited through an online panel marketplace (Lucid), managed by the market research firm. Respondents who participated in the marketplace received different incentives depending on how they were recruited (e.g., cash, cash equivalents, reward points). This sample size was chosen to ensure regional subgroups had sufficient respondents for analysis.

Informed consent was obtained from all individual participants included in the study and their parent or legal guardian in the case of youth under 16 years of age. Prior to participating, respondents indicated their consent by selecting a “Yes” response confirming their understanding and consent. Youth were recruited through their parents and also provided informed consent. Respondent information was fully anonymized before data collection, aggregation, and analysis.

The final sample was weighted to match 2021 Canadian census data from Statistics Canada. Weighted data was used throughout the analysis. Respondents who completed the survey in unrealistically short times were excluded to enhance data validity.

### 2.2 Questionnaire design

The bilingual (English and French) questionnaire was collaboratively developed by Abacus Data and the Public Health Agency of Canada. The instrument was designed to measure socio-demographic characteristics, perceived risk, PPM use, beliefs about PPM effectiveness, and determinants of PPM use. Items related to perceived risk included participants’ perceived risk of severe illness from RIDs, perceived level of disease circulation in their community, and worry about others not taking preventive measures. The instrument required a response to each question in order to be included in the final data set. There was, however, the option to indicate “prefer not to answer”.

The potential determinants of PPM use that were investigated included trust in public health information, perception of PPMs role in protecting vulnerable populations, and the influence of social norms, such as seeing others wearing masks. PPM use was measured by asking respondents to rate the frequency of engaging in six specific PPMs over the past 3 months: wearing masks in indoor public settings, staying home when sick (only asked of a subset reporting recent illness), cleaning hands regularly, covering coughs and sneezes, cleaning and disinfecting high-touch surfaces, and improving ventilation.

Respondents rated PPM effectiveness based on the perceived ability in reducing the spread of RIDs. Barriers to PPM use were examined for respondents who reported infrequent use of specific PPMs. All variables were measured using 10-point (range: 1 = not at all to 10 = extremely), 5-point (range: 1 = never to 5 = always), or 4-point (range: 1 = not effective to 4 = very effective) scales, as appropriate. To support the regression model creation, several demographic variables were re-coded to enable direct relationships with PPMs. Gender was analyzed as a binary (male = 2, female = 1) and employment status was broken into three dichotomous variables for working, retired and student status (where yes = 1 and no = 0). The survey was pretested with 25 respondents. The complete questionnaire is available in the full study report (18).

### 2.3 Data analysis

Data were analyzed using the Statistical Package for the Social Sciences (SPSS) software, version 29. Descriptive statistics were used to summarize socio-demographic characteristics as well as attitudes, perceptions, and behaviors of the sample.

Inferential statistics were used to identify univariate differences in PPM use based on demographic characteristics age and vaccination within the sample. Differences between subgroups were identified using a non-parametric statistical test (Mann–Whitney U) in SPSS at the *p*-value <0.05 significance level within the regression model results only. These are to be considered internally valid comparisons and not generalizable to the population as no estimate of sampling error can be calculated on a non-probability sample and the results cannot be assigned a margin of error.

Multiple linear regression analyses were conducted to examine factors influencing individual PPM use. Separate regression models were created for the six PPMs (i.e., the dependent variables) which estimated the role of different variables in accounting for the variation in PPM use. Independent variables included perceived effectiveness, an index of personal risk of severe consequences of infection across RIDs, local circulation of RIDs, trust in public health information, attitudes toward PPMs, personal health factors (e.g., vaccination status), and demographics. Multiple linear regression was used, treating the ordered outcome variables as approximately continuous, as parametric methods such as linear regression have been shown to be relatively robust when applied to Likert-type data with multiple ordered response categories (19). Standardized beta coefficients were reported to facilitate comparison across variables, with significance levels set at *p* < 0.05. Missing data (such as “prefer not to answer” responses) were not included in this analysis. Similarly, the model for the PPM “staying home when sick” was limited to those who reported symptoms of a respiratory illness during the previous 3 months.

A stepwise variable selection process was used to select relevant predictor variables and reduce overfitting. Demographic variables were entered first, followed iteratively by groups of predictor variables. Where collinearity identified, one of the collinear variables was excluded from the final model based on epidemiological relevance. Model assumptions were assessed using residual and normal probability plots, with no serious violations identified. Variance inflation factors (VIFs) were all below 3 (maximum = 2.83), indicating no problematic multicollinearity. The final models included independent variables selected based on theoretical relevance and statistical considerations, including evidence of association with the outcome.

### 2.4 Human ethics declaration

All procedures involving human participants were in accordance with the ethical standards of the institutional and/or national research committee (Public Opinion Research Directorate for the Government of Canada POR 085-23 and the Canadian Research Insights Council Reg #20240205-AB857) in accordance with the Standards for the Conduct of Government of Canada Public Opinion Research (28), Canadian Code of Market, Opinion, and Social Research and Data Analytics (30), as well as the internationally recognized European Society for Opinion and Marketing Research (ESOMAR) standards (29) and ISO 20252:2019 standard. The project was approved by Public Opinion Research Directorate at Public Services and Procurement Canada, and the Public Opinion Research and Analysis Unit at Health Canada.

Health Canada/Public Health Agency of Canada’s Privacy Management Division has reviewed the methodology (HC-CP-2023-000024) and research instruments (PHAC-PP-2023-000071) with respect to this research project.

This article does not contain any studies with animals performed by any of the authors.

## 3 Results

### 3.1 Demographic characteristics

Of the 9,600 individuals who viewed the survey on the marketplace, 7,906 responded to the survey. Of those who responded, 1,295 surveys were disqualified due to sufficiently met quotas and incomplete responses. A total of 6,611 participants across Canada completed the survey and were included in this analysis.

The respondent profile closely resembles the 2021 Canadian census. For example, 50% identified as male, 50% identified as female and less than 1% indicated another option. Respondents were similarly distributed by age with 7% of respondents aged 12 to 17, 27% aged 18 to 34, 29% aged 35 to 54, and 37% aged 55 and over.

A detailed description of the participants is included in the Supplementary Tables S1, S2.

#### 3.1.1 Personal health factors

Underlying health conditions seemed relatively common as 39% of responding adults (aged 18+) and 10% of youth (ages 12–17) indicated that they were at higher risk of severe illness or outcomes because of an underlying health condition (see Supplementary Table S6). Among respondents, 13% had symptoms of a respiratory illness in the 2 weeks prior to completing the survey (see Supplementary Table S5). Almost half (47%) had received their fall 2023 COVID-19 booster, while 86% reported receiving at least one COVID-19 vaccine in their lifetime (see Supplementary Table S4).

### 3.2 Reported use of personal protective measures

As shown in Table 1, reported PPM use in the 3 months prior to the survey varied by measure. The most commonly reported practices included covering coughs/sneezes (84% always/often), regular hand cleaning (83% always/often), and those recently sick staying home while sick (62% always/often). Less common practices included cleaning and disinfecting high-touch surfaces (54% always/often) and improving ventilation (50% always/often). The least frequently reported PPM was mask-wearing in indoor public settings (24% always/often). Note that only those who reported having symptoms of a respiratory illness within the past 3 months (n = 3,078) were asked if they had stayed home when sick.

**Table 1:** Frequency of reported use of personal protective measures (PPMs).

Personal protective measure	Always		Often		Sometimes		Rarely		Never		Not applicable	
	%	#	%	#	%	#	%	#	%	#	%	#
Covering coughs and sneezes with your elbow or a tissue	64%	4,248	20%	1,369	9%	569	4%	221	1%	96	2%	108
Cleaning your hands regularly	55%	3,676	28%	1873	11%	701	3%	214	2%	106	1%	41
Staying home when sick	35%	1,079	27%	850	20%	603	8%	247	7%	210	3%	89
Cleaning and disinfecting high-touch surfaces and objects	25%	1,620	29%	1926	25%	1,699	13%	839	7%	458	1%	69
Improving indoor ventilation	23%	1,484	26%	1740	26%	1739	13%	857	9%	612	3%	179
Wearing a mask when in indoor public settings	11%	696	13%	873	23%	1,508	20%	1,326	30%	2009	3%	199

Q25. Within the past 3 months, how often have you taken the following personal protective measures (PPMs)? Base (*n*) = 6,611 [if sick less than 3 months ago *n* = 3,078].

#### 3.2.1 Reasons cited for not using PPMs

As illustrated in Figure 1, several themes emerged to explain less frequent PPM use (i.e., sometimes/rarely/never), including a lack of a requirement/mandate, not having an option to work from home while sick, forgetting to bring hand sanitizer, lacking resources to improve air circulation, and not believing that adopting the PPM would make a difference.

**Figure 1:**
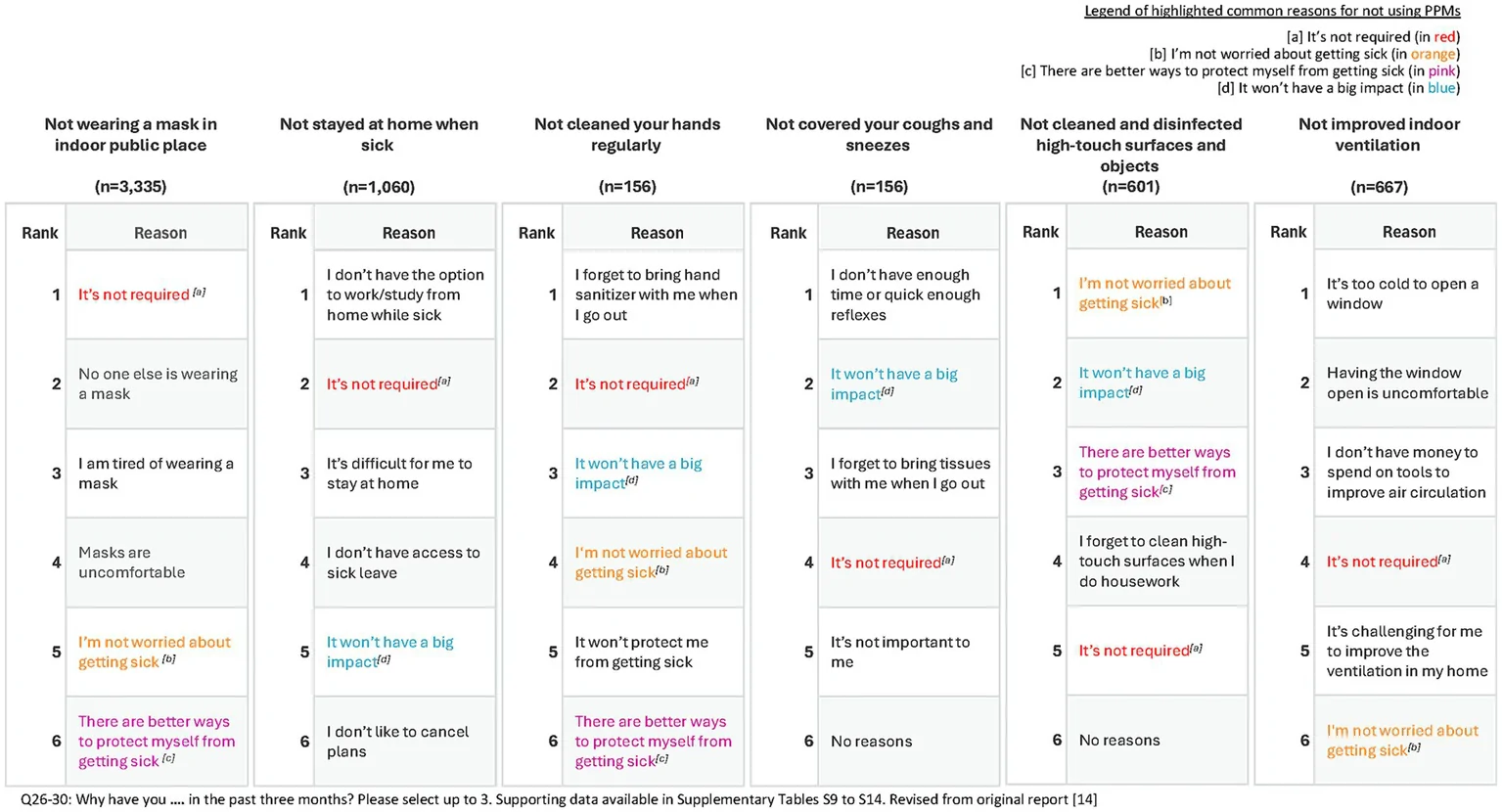
Ranked order of responses for reasons not to use by PPM. Q26-30: Why have you… in the past 3 months? Please select up to 3. Supporting data available in Supplementary Tables S9–S14. Revised from original report (14).

#### 3.2.2 Trust in public health information received from the Government of Canada

Three in four (77%) respondents trusted the public health information they receive from the Government of Canada.

### 3.3 Perceived effectiveness of PPMs in reducing spread of RIDs

All six of the PPMs were perceived as at least somewhat effective in reducing the spread of RIDs by a large majority of respondents. As shown in Table 2, staying home when sick and regular handwashing were deemed the most effective measures, with 95% of respondents considering them at least somewhat effective, followed closely by covering coughs and sneezes (93% very/somewhat effective). Despite ranking the lowest for perceived effectiveness of all PPMs listed, wearing masks in indoor public settings was still considered effective by a large majority (80% very/somewhat effective).

**Table 2:** Perceived effectiveness of PPMs.

Personal protective measure	Very + somewhat effective (NET)		Very effective		Somewhat effective		Somewhat not effective		Not effective	
	%	#	%	#	%	#	%	#	%	#
Staying home when sick	95%	6,287	72%	4,830	22%	1,457	4%	241	1%	83
Cleaning your hands regularly	95%	6,288	67%	4,490	27%	1798	4%	246	1%	77
Covering coughs and sneezes with your elbow or a tissue	93%	6,146	53%	3,518	40%	2,628	6%	369	2%	96
Cleaning and disinfecting high-touch surfaces and objects (for example, phones, doorknobs)	90%	5,967	49%	3,233	41%	2,734	8%	511	2%	133
Wearing a mask when in indoor public settings (e.g., transit, stores, concerts)	80%	5,283	39%	2,536	41%	2,747	12%	816	8%	512
Improving indoor ventilation (for example, opening windows and doors when possible and/or using a portable air purifier)	88%	5,844	37%	2,444	51%	3,400	9%	594	3%	173

Q33. How effective do you think each of the following personal protective measures are in reducing the spread of respiratory infectious diseases? Base (*n*) = 6,611.

### 3.4 Personal risk of infection and severe outcomes

In February 2024, 37% of respondents perceived the level of circulation of RIDs in their communities to be low or very low, 14% perceived it to be high or very high, and 16% were unsure. Almost two in three (65%) were worried that others are not taking steps to avoid getting or spreading RIDs.

The risk of personally having a severe consequence as a result of being infected from an RID did not vary by disease evaluated (see Supplementary Table S7). About three in ten (31–32%) respondents perceived their risk of severe outcomes due to any of the three RIDs (COVID-19, RSV and the flu) to be very low (1 rating on a 1–10 scale), while one in ten (10–11%) felt they would be at high risk (8–10 rating). Three in ten (28%) often or sometimes spent time with someone else at higher risk of a severe illness due to an underlying health condition or their age (see Supplementary Table S8).

### 3.5 Attitudes towards PPM use

Four in five (81%) respondents agreed that following public health advice by using PPMs is an effective way to protect vulnerable people and almost three in five (59%) indicated that they use PPMs when they are concerned about getting sick themselves, as shown in Table 3. Three in five (60%) report being more likely to wear a mask if they see others around them using one.

**Table 3:** Attitudes towards PPM use.

Statement	% Agree/somewhat agree		Agree		Somewhat agree		Somewhat disagree		Disagree		Not sure	
	%	#	%	#	%	#	%	#	%	#	%	#
Following public health advice by using personal protective measures is an effective way to protect the vulnerable people in my community	81%	5,374	39%	2,627	42%	2,747	8%	542	6%	396	5%	299
I trust the public health information I receive from the Government of Canada	77%	5,075	38%	2,512	39%	2,563	10%	661	9%	626	4%	248
Using personal protective measures is important to me	71%	4,692	30%	1961	41%	2,731	15%	1,003	10%	665	4%	251
I’m more likely to use a mask when I see others around me using one	60%	3,952	23%	1,493	37%	2,459	18%	1,144	18%	1,206	5%	309
I use personal protective measures (e.g., wearing a mask, improving indoor ventilation) because I’m concerned about getting sick	59%	3,862	24%	1,576	35%	2,286	19%	1,241	18%	1,257	4%	251

Q35/Q37: How much do you agree or disagree with each of the following? Base (*n*) = 6,611.

### 3.6 Factors influencing variations of PPM use

The regression models for each PPM had varying levels of explanatory power with the adjusted R-squared values for the regression models ranging from 0.216 for staying home when sick to 0.407 for wearing a mask in indoor public settings.

Figure 2 provides a summary of the six regression models, each corresponding to a different dependent variable. Standardized Beta coefficients (β) are reported, ranging from −1 to +1, to allow for comparison of the relative impact of the independent variables across models. (Further details are available in Supplementary Table S3).

**Figure 2:**
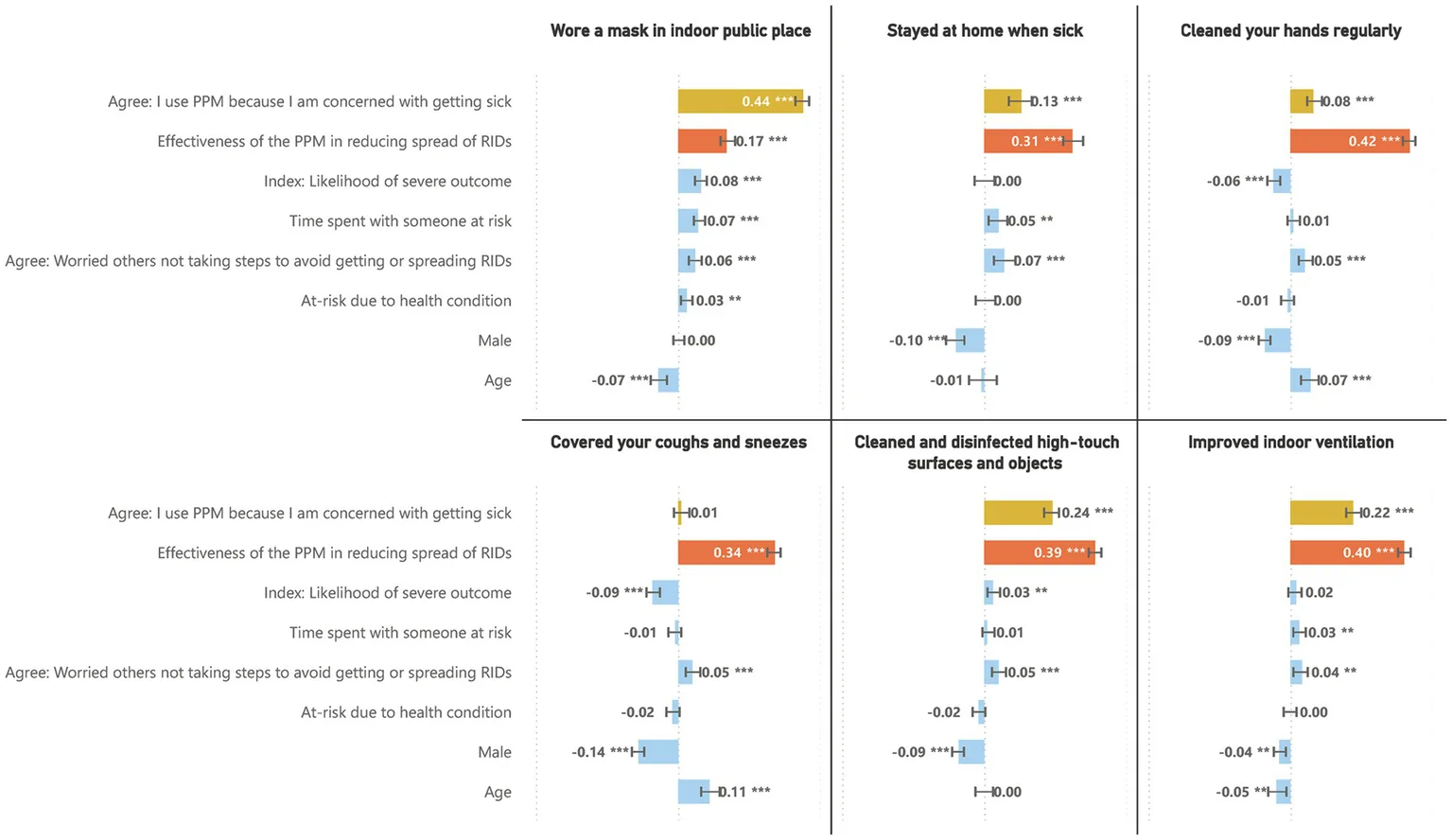
Regression factors influencing individual PPM use. Regression results further highlighted in Supplementary Table S3. Coefficients are Beta values, which allow more easy comparisons given that the values are standardized (−1 to 1). The confidence intervals presented should be considered internally valid comparisons only and not generalizable to the population. No estimate of sampling error can be calculated on a non-probability sample and the results cannot be assigned a margin of error. Factors impacting 4 or more PPMS shown. Dependent variable Q25: Within the past 3 months, how often have you taken the following personal protective measures (PPMs)? Base: *n* = 6,611. Significance: * *p* < 0.05; ** *p* < 0.01; *** *p* < 0.001. Revised from original report (14).

Overall, the results from the multiple linear regression models reveal that the strongest factor associated with reported PPM use among respondents was the belief that the PPM was effective. For every PPM, perceived effectiveness in reducing the spread of RIDs was significantly associated with use of the PPM (*β* = 0.17 to 0.42, *p* < 0.001).

Several additional variables were positively associated with using all or most of the PPMs.

On average, respondents who expressed concern that others were not taking adequate steps to prevent the spread of RIDs were more likely to use all forms of PPMs compared to those who were not worried (*β* = 0.04 to 0.07, *p* < 0.01). Similarly, individuals who reported using PPMs due to concern with getting sick were significantly more likely to engage in nearly all PPM behaviors—particularly mask-wearing (*β* = 0.44, *p* < 0.001), cleaning and disinfecting surfaces (*β* = 0.24, *p* < 0.001), and improving ventilation (*β* = 0.22, *p* < 0.001) —though this association did not extend to covering coughs and sneezes. A negative relationship between gender and PPM use suggests that females were generally more likely to engage in PPM use than males (*β* = −0.04 to −0.14, *p* < 0.001), except for mask-wearing, where no notable gender difference was found (*β* = 0).

In addition to these broad trends, some factors were only associated with the use of a specific PPM among the responding sample. For example, age influenced behavior with older respondents more likely than younger individuals to wash their hands and cover coughs and sneezes (*β* = 0.11, *p* < 0.001) but were less likely to wear masks in indoor public places (*β* = −0.07, *p* < 0.001) or improve ventilation (*β* = −0.05, *p* < 0.01).

Occupational status also revealed distinct patterns: compared to other occupations, students were less likely to report mask-wearing (*β* − 0.05, *p* < 0.001), disinfecting (*β* − 0.08, *p* < 0.001), and improving ventilation (*β* − 0.06, *p* < 0.001) but more likely to cover coughs and sneezes (*β* 0.03, *p* < 0.05). Those who were employed were more likely to report consistent hand hygiene (*β* 0.03, *p* < 0.05) and covering coughs and sneezes (*β* 0.06, *p* < 0.01), while retired individuals were more likely to stay home when sick (*β* 0.11, *p* < 0.001) but less likely to disinfect surfaces (*β* − 0.05, *p* < 0.01).

Being at high risk due to a health condition and having recently been ill were both positively associated with mask-wearing in public indoor spaces (each *β* 0.03, *p* < 0.001), but not with other PPMs. Vaccinated individuals were also more likely to wear masks indoors (*β* 0.03, *p* < 0.05) but were less likely to report staying home when sick (*β* − 0.04, *p* < 0.05); vaccination status was not significantly associated with the other PPMs.

When looking at perceptions of risk, individuals who perceived themselves as being at higher risk for severe outcomes from RIDs were more likely to wear masks in indoor public spaces (*β* = 0.08, *p* < 0.001) and clean and disinfect surfaces and objects (*β* = 0.03, *p* < 0.01). However, this perceived risk was not consistently associated with the use of other PPMs and was, in fact, negatively associated with handwashing (*β* = −0.06, *p* < 0.001) and covering coughs and sneezes (*β* = −0.09, *p* < 0.001).

Likewise, perceptions of community-level risk also played a role. Those who believed there was a high level of RID circulation in their area were more likely to wear masks (*β* = 0.05, *p* < 0.001) and stay home when sick (*β* = 0.04, *p* < 0.05) but were less likely to improve ventilation (*β* = −0.03, *p* < 0.05). Similar associations were observed for respondents who spent time with individuals at risk for severe illness—this was positively associated with mask-wearing (*β* = 0.07, *p* < 0.001), staying home when sick (*β* = 0.05, *p* < 0.01), and improving ventilation (*β* = 0.03, *p* < 0.01).

Slightly different PPMs were associated with believing that following public health advice helps protect vulnerable individuals – this belief was positively associated with staying home when sick (*β* 0.06, *p* < 0.05), hand hygiene (*β* 0.10, *p* < 0.001), and covering coughs and sneezes (*β* 0.14, *p* < 0.001).

Lastly, trust in public health information from the Government of Canada was negatively associated with cleaning and disinfecting surfaces (*β* − 0.03, *p* < 0.05) and showed no meaningful association with other PPMs.

## 4 Discussion

This study provides a comprehensive overview of PPM use in Canada following the acute phase of the COVID-19 pandemic. Although this study used a non-probability study sample, the resulting respondent profile closely reflects the 2021 Canadian Census. While the results cannot be extrapolated to the broader population, the findings indicate that while many Canadians may have continued to engage in certain protective behaviors, other measures were less commonly adopted at the time of the survey.

The most commonly reported PPM practices were covering coughs and sneezes and cleaning hands regularly, with over 80% of respondents reporting consistent use. This is consistent with previous literature highlighting these behaviors as ingrained public health habits across multiple contexts (14, 15, 20, 21).

In contrast, mask-wearing in indoor public settings had the lowest reported use (24%), suggesting that as public mandates relaxed, voluntary mask use may have significantly declined in Canada. This aligns with research in the US that found the removal of mandates often leads to decreased adherence to protective behaviors (22). Additionally, social cues seemed to have some impact on masking behaviors.

Perceived effectiveness of a PPM was the strongest and most consistent factor associated with reported use within the fitted regression model across all but one of the assessed PPMs. This supports the Theory of Planned Behavior, which emphasizes that beliefs about outcomes play a crucial role in shaping behavior (23, 24). Individuals who believed a measure was effective were significantly more likely to report using it, highlighting the need for clear, evidence-based public messaging about the effectiveness of protective actions.

Demographic variables such as gender and age also influenced reported PPM use among survey respondents. Women and older adults were more likely to consistently reported use of most PPMs, except for mask-wearing and improving ventilation. These findings are in line with prior studies which reported higher adherence with public health recommendations among women and older individuals (8, 25). Moreover, respondents at higher risk for severe outcomes from RIDs, or who spent time with vulnerable populations, were more likely to report engaging in behaviors such as mask-wearing and staying home when sick—pointing to the potential role of both personal and interpersonal risk perceptions in shaping protective behavior.

The regression results also indicated that trust in public health information from the Government of Canada did not have significant influence on reported PPM use among survey respondents. This finding was somewhat unexpected as previous studies typically find that trust in governments and the information they provide is linked to PPM adoption (7, 8, 10). This finding might suggest that after the acute phase of an epidemic, trusted advice alone is no longer sufficient to drive behavior and that information must be coupled with meaningful messaging around personal relevance and effectiveness.

Reasons cited for not using PPMs were multifaceted. A lack of mandates, practical challenges (e.g., inability to work from home, lack of resources), and skepticism about PPM effectiveness were the most commonly cited reasons for non-use among survey respondents. As such, potential avenues for facilitating protective measures could include addressing common barriers, creating environments supportive of PPM adoption, and clear communication about PPM effectiveness and recommendations.

Future research could continue investigating PPM use and drivers for use during interpandemic periods and during cold and flu seasons. This could contribute to understanding evolving patterns, any important differences in sub-groups, and how to best support effective protective behaviors. Additionally, research on effective communications and supports for promoting and increasing PPM use during non-crisis situations is valuable and can inform public health before, during, and after any future pandemic or outbreak. This could include studying communications that leverage existing adopted behaviors and social cues to promote the adoption of other PPMs.

## 5 Contributions to knowledge

This study contributes to the literature by examining drivers and barriers to PPM use after the acute period of the pandemic, moving beyond the COVID-19-specific focus of recent research and focusing also on seasonal influenza and RSV. While much of the pandemic research focused on adherence during peak transmission periods, this study sheds light on behavior patterns and drivers in a more normalized context, offering insights for preparedness strategies during seasonal outbreaks or future pandemics.

## 6 Limitations

The survey results cannot be generalized to the broader population in Canada, as the sampling method does not guarantee that the sample accurately represents the target population with a measurable margin of error. These limitations may have an impact on the interpretation of associations. In addition, current evidence also shows that due to social desirability bias, individuals tend to over report their PPM adherence, with self-reported adherence rates reaching up to 99%, significantly higher than observed rates, which are sometimes as low as 46% in healthcare settings (26, 27). Given the survey results are self-reported within the survey tool, the relationships between some variables, such as “concern about getting sick” and “PPM use,” could be inflated due to common-method variance.

There is also a possibility of non-response bias, which is introduced because certain types of individuals may be more or less likely to respond to the survey. The survey does not, for example, include members of the population who do not have access to the Internet. In addition, there are some groups within the population that are systemically less likely to answer surveys. The paid incentive for the panel assists in engaging with these underrepresented groups, however adds an opt-in bias as similarly, certain groups are more likely to participate in research panels. The 12 to 17 year old sample, which was mostly recruited through parents, may also be impacted by the decision of the parent to allow/encourage their child to complete the survey.

Finally, reported PPM use was not assessed in relation to local respiratory disease surveillance data, limiting the ability to assess how PPM use may differ during periods of higher local disease activity or an active outbreak.

## 7 Conclusion

In conclusion, this study highlights that while certain personal protective behaviors remain commonly reported in Canada, their adoption and perceptions regarding their effectiveness varies. These findings can help inform interventions to address barriers to PPM use and communications strategies for both emergency responses during pandemics and also for RIDs such as RSV and seasonal influenzas. The results suggest that public health communications emphasizing the effectiveness of these interventions to reduce the spread of RIDs and the potential individual risks of infection may be an effective strategy to promote PPM use.

## Data Availability

Publicly available datasets were analyzed in this study. This data can be found at: Library and Archives Canada—Public Opinion Research Database https://epe.bac-lac.gc.ca/100/200/301/pwgsc-tpsgc/por-ef/public_health_agency_canada/2024/085-23-e/index.html?nodisclaimer=1.
